# Prevention of selenite-induced cataractogenesis by rutin in Wistar rats

**Published:** 2009-12-04

**Authors:** M. Isai, M. Sakthivel, E. Ramesh, P.A. Thomas, P. Geraldine

**Affiliations:** 1 Department of Animal Science, School of Life Sciences, Bharathidasan University, Tamil Nadu, India; 2 Institute of Ophthalmology, Joseph Eye Hospital, Tamil Nadu, India

## Abstract

**Purpose:**

To investigate whether rutin retards selenite-induced cataractogenesis in Wistar rat pups.

**Methods:**

On postpartum day ten, Group I rat pups received an intraperitoneal injection of saline. Group II and III rat pups received a subcutaneous injection of sodium selenite. Group III also received an intraperitoneal injection of rutin once daily on postpartum days 9–14. Both eyes of each pup were examined from day 16 up to postpartum day 30. After sacriﬁce, extricated pup lenses were analyzed for mean activities of catalase, superoxide dismutase, glutathione peroxidase, glutathione S-transferase, and glutathione reductase. In addition, the mean concentrations of reduced glutathione (GSH) and of malondialdehyde were analyzed in samples of lenses and hemolysate.

**Results:**

There was dense lenticular opaciﬁcation in all of Group II, minimal opaciﬁcation in 33.3% of Group III, no opaciﬁcation in 66.7% of Group III, and no opaciﬁcation in Group I. Signiﬁcantly lower mean activities of lenticular antioxidant enzymes were noted in Group II, compared to Group I and III. Significantly lower mean concentrations of GSH and higher mean concentrations of malondialdehyde were noted in samples of hemolysate and lens from Group II, compared to the values in Group I and III.

**Conclusion:**

Rutin prevents experimental selenite-induced cataractogenesis in rat pups, possibly by preventing depletion of antioxidant enzymes and of GSH, and by inhibiting lipid peroxidation.

## Introduction

Cataracts are the most common cause of blindness worldwide, and their incidence is likely to increase as individuals in the current generation grow older [1]. Currently, surgery is the mainstay of the management of cataracts, but there continues to be a backlog in the services provided in many parts of the world [2]. Delaying the onset of cataracts by pharmacological means may help to bridge the gap between the high incidence of cataract blindness and the provision of surgical treatment. Various experimental models have been used to study the etiology of cataracts and to investigate different therapeutic modalities.

There is a large body of evidence implicating oxidative stress and radical oxygen species in the mechanism of cataractogenesis [3,4]. The selenite cataract is a rapidly induced, convenient model for the study of senile nuclear cataractogenesis [5]. Various compounds have been shown by experimental studies to prevent selenite-induced cataractogenesis, including resveratrol [6], melatonin [7], acetyl-L-carnitine [8,9], ellagic acid [10], soya bean [11], extract of *Pleurotus ostreatus* [12], L-cysteine [13], N-acetylcysteine [14], and onion juice [15].

Mushrooms constitute an integral part of the normal human diet. They are considered valuable health foods, since they are low in calories, fats, and essential fatty acids, and high in vegetable proteins, vitamins, and minerals [16,17]. Mushrooms reportedly contain relatively large amounts of vitamins A and C and of β-carotene, all of which have protective effects because of their antioxidant properties [18]. Mushrooms also contain many phenols, which are very efficient scavengers of peroxy radicals [18], and are reported to be excellent antioxidants and synergists that are not mutagenic [19].

We have previously shown that an ethanolic extract of the oyster mushroom, *Pleurotus ostreatus,* exerts an anticataractogenic effect in an animal model [12]. Interestingly, a high concentration of rutin (31.2 mg/100 g) has been observed in *P. ostreatus* [20]. Rutin (quercetin-3-*o*-β-rhamnosylglucose) is a flavone glycoside that is abundantly present in herbs and plant foods. It has been reported to scavenge free radicals, to lower hepatic and blood cholesterol levels, and to exhibit antiplatelet activity [21,22]. In addition, it has shown significant antioxidant activity in a liposomal model reaction [23]. Nagasawa et al. [24] have also shown that 0.2% of G-rutin (a rutin–glucose derivative), given in a 20% casein diet for a period of one month to streptozotocin-induced diabetic rats, decreased thiobarbituric acid reactive substance (TBARS) in kidneys. Therefore, we reasoned that rutin possibly plays a major role in the anticataractogenic effect of the ethanolic extract of *P. ostreatus.* This paper describes an attempt to test this hypothesis in an experimental model of selenite-induced cataractogenesis.

## Methods

Nine-day-old rat pups (Wistar strain) were used in this study. The pups were housed with parents in large spacious cages, and the parents were given food and water ad libitum. The animal room was well ventilated, and a regular 12 h:12 h light-dark cycle was maintained throughout the experimental period. These animals were used in accordance with institutional guidelines and with the Association for Research in Vision and Ophthalmology Statement for the Use of Animals in Research. The rat pups were divided into one control group and two experimental groups, each group comprising 12 pups:

1. Group I, which received only saline (control),
2. Group II, which received selenite alone (cataract-untreated), and
3. Group III, which received selenite and rutin hydrate (cataract-treated).

Each rat pup in Groups II and III received a single subcutaneous (s.c.) injection of sodium selenite (19 µmol/kg body weight) on postpartum day ten. In addition, pups in Group III received intraperitoneal (i.p.) injections (175 mg/kg body weight) of rutin hydrate (Sigma Chemical Co., St. Louis, MO). The ﬁrst dose of rutin was administered one day prior to the selenite injection, and the rutin injection was repeated once daily for ﬁve consecutive days thereafter.

### Morphological examination of rat pup lenses

When the rat pups ﬁrst opened their eyes (approximately 16 days after birth), a slit-lamp biomicroscopic examination was performed on each eye of each rat pup to provide a morphological evaluation of any lenticular opaciﬁcation. Prior to performing the examination, mydriasis was achieved by using a topical ophthalmic solution containing tropicamide with phenylephrine (Maxdil Plus, Hi-Care Pharma, Chennai, India). One drop of the solution was instilled every 30 min for 2 h into each eye of each rat pup, with the animals being kept in a dark room. After 2 h, the eyes were viewed by a slit-lamp biomicroscope at 12× magniﬁcation. At the end of the experimental period (postpartum day 30), the degree of lenticular opaciﬁcation was graded and photographed. The degree of opaciﬁcation was graded as follows:

0=normal transparent lens
+=initial sign of nuclear opacity involving tiny scatters
++=partial nuclear opacity
+++=mature nuclear opacity

### Biochemical analysis of lenses and blood samples from rat pups

#### Preparation of hemolysate for analysis

After morphological examination on day 30, blood was drawn from each rat pup in the three groups. From each blood sample, serum was separated, and a hemolysate was prepared according to the procedure of Dodge et al. [25] as modified by Quist [26]. Sample preparation was performed at 4 °C, and all the samples were stored at −70 °C until analysis.

#### Preparation of lenses for analysis

After blood was drawn from the rat pups, the animals were anaesthetized by diethylether and then sacrificed by cervical dislocation. The lenses were then removed by dissection. The lenses from each group of rat pups were homogenized in ten times their mass of 50 mM phosphate buffer (pH 7.2), and centrifuged at 12,000 rpm for 15 min at 4 °C. The supernatant obtained was stored at −70 °C in aliquots until used for the analysis of enzyme activities. To calculate the speciﬁc enzyme activity, protein in each sample was estimated by the method of Bradford [27] by using bovine serum albumin as a standard.

### Analysis of antioxidant enzymes

#### Catalase

Catalase (CAT) activity was determined by the method of Sinha [28]. In this test, dichromatic acetic acid is reduced to chromic acetate when heated in the presence of hydrogen peroxide (H_2_O_2_), with the formation of perchloric acid as an unstable intermediate. In the test, the green color developed was read at 590 nm against blank on a spectrophotometer. The activity of catalase was expressed as units/mg protein (one unit was the amount of enzyme that utilized 1 mmol of H_2_O_2_/min).

#### Superoxide dismutase

Superoxide dismutase (SOD) activity was determined by the method of Marklund and Marklund [29]. In this test, the degree of inhibition of pyrogallol auto-oxidation by the supernatant of the lens homogenate was measured. The change in absorbance was read at 470 nm against blank every 60 s for 3 min on a spectrophotometer. The enzyme activity was expressed as units/min/mg protein (one unit was considered to be the amount of enzyme that inhibited pyrogallol auto-oxidation by 50%).

#### Glutathione peroxidase

The activity of glutathione peroxidase (Gpx) was determined essentially as described by Rotruck et al. [30]. The principle of this method is that the rate of glutathione oxidation by H_2_O_2_, as catalyzed by the Gpx present in the supernatant, is determined, and the color that develops is read against a reagent blank at 412 nm on a spectrophotometer. In the test, the enzyme activity was expressed as units/mg protein (one unit was the amount of enzyme that converted 1 µmol of reduced glutathione [GSH] to the oxidized form of glutathione [GSSH] in the presence of H_2_O_2_/min).

#### Glutathione reductase

This enzyme glutathione reductase (GR), which utilizes nicotinamide adenine dinucleotide phosphate (NADPH) to convert oxidized glutathione to its reduced form, was assayed by the method of Stall et al. [31]. The change in absorbance was read at 340 nm for 2 min at intervals of 30 seconds in a UV-spectrophotometer. The activity of glutathione reductase was expressed as nmol of NADPH oxidized/min/mg protein.

#### Glutathione S-Transferase

The activity of glutathione S-transferase (GST)was determined by the method of Habig and Jacoby [32]. The conjugation of GSH with 1 chloro, 2-4 dinitrobenzene (CDNB), a hydrophilic substrate, was observed spectrophotometrically at 340 nm to measure the activity of GST; one unit of GST was defined as the amount of enzyme required to conjugate 1 μmol of CDNB with GSH/min.

### Estimation of reduced glutathione and malondialdehyde content in lenses and hemolysate

The GSH content was estimated by the method of Moron et al. [33]. Each lens was homogenized in 1 ml of 0.1 M phosphate buffer, and was centrifuged at 5,000 rpm for 15 min at 4 °C. To the supernatant of the lenticular homogenate or hemolysate, 10% trichloroacetic acid (0.5 ml) was added, followed by recentrifugation. To the protein-free supernatant, 4 ml of 0.3 M Na_2_HPO_4_ (pH 8.0) and 0.5 ml of 0.04% (wt/vol) 5,5-dithiobis-2-nitrobenzoic acid were added. The absorbance of the resulting yellow color was read spectrophotometrically at 412 nm. A parallel standard was also maintained. The results were expressed in µmol/g wet weight for the lenses and nmoles/g hemoglobin (Hb) for hemolysate.

The extent of lipid peroxidation was determined by the method of Ohkawa et al. [34]. Briefly, 0.2 ml of 8.1% sodium dodecyl sulphate, 1.5 ml of 20% acetic acid (pH 3.5), and 1.5 ml of 0.81% thiobarbituric acid aqueous solution were added in succession. To this reaction mixture, 0.2 ml of the tissue sample (lenticular homogenate or hemolysate) were added. The mixture was then heated in boiling water for 60 min. After cooling to room temperature, 5 ml of butanol:pyridine (15:1 v/v) solution were added. The mixture was then centrifuged at 5,000 rpm for 15 min. The upper organic layer was separated, and the intensity of the resulting pink color was read at 532 nm. Tetramethoxypropane was used as an external standard. The level of lipid peroxide was expressed as nmoles of MDA formed/g wet weight for lenses, and nmoles of MDA formed/g Hb for hemolysate.

### Statistical analysis

The values are expressed as the mean±SD for the 12 pups in each group. Differences between groups were assessed by one-way analysis of variance (ANOVA) using the Statistical Package for Social Sciences (SPSS) software package for Windows (version 16.0). Post hoc testing was performed for intergroup comparisons using the least significant difference (LSD) test, and the chi-square test was applied wherever relevant. A value corresponding to p<0.05 was deemed to be statistically significant.

## Results

### Morphological examination to determine lenticular opacification in rat pups

**Figure 1:**
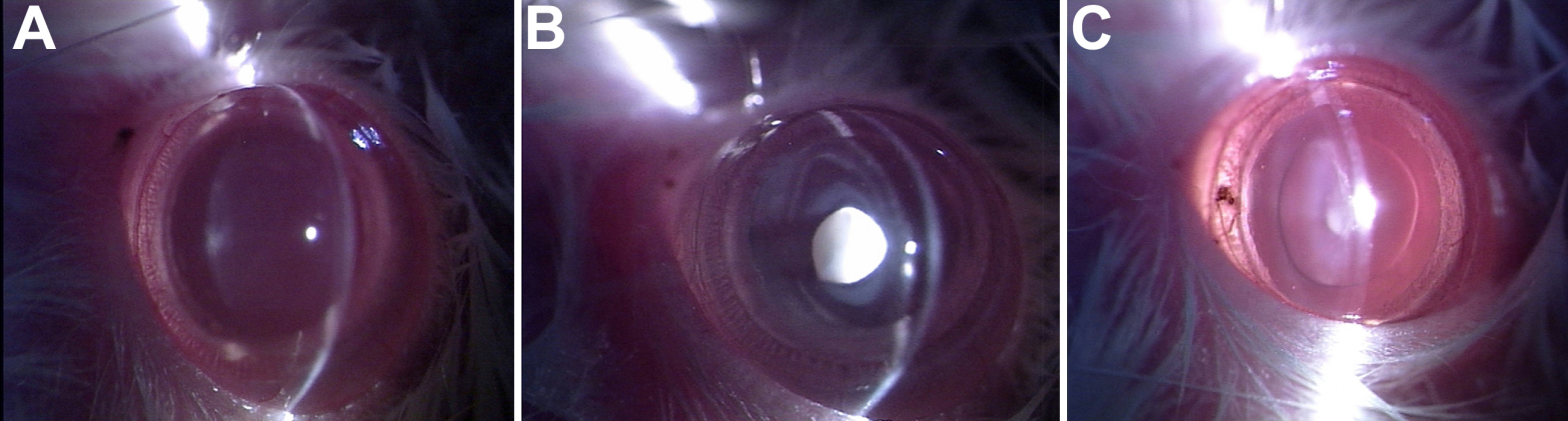
Slit-lamp appearance of lenses in eyes of 16-day-old Wistar rat pups. **A**: Lenses in normal (Group I) pup eyes have all remained transparent (grade 0 opacity). **B**: Lenses in cataract-untreated (Group II) eyes have all become densely opaque (grade +++ opacity). **C**: Lenses in some cataract-treated (Group III) pup eyes have become mildly opaque (grade + opacity).

Morphological examination of both eyes of each rat pup was done by slit-lamp examination on the 30th day after birth. All 12 rat pups in Group II (which received s.c. injections of sodium selenite) exhibited dense opacification of the lenses (grade +++ opacity). In contrast, only 4 of 12 (33.3%) rat pups in Group III (which received i.p. injections of rutin hydrate, along with sodium selenite) exhibited mild lenticular opacification (grade +), with the lenses of the other eight pups appearing normal (grade 0). All 12 rat pups in Group I (which received normal saline as the control) exhibited complete transparency (grade 0) of the lens (Figure 1, Table 1). The difference between the percentages of Group II and of Group III exhibiting lenticular opacification was statistically significant (χ2 [ degree of freedom=1]=4.0; p<0.05).

**Table 1:** Morphological examination of lenses of rat pups.

**Experimental groups**	**Number of pups**	**Number of pups with different degrees of lenticular opacification**				**Number of pups in which lenticular opacification occurred**
		**0**	**+**	**++**	**+++**	
Group I (normal)	12	12	-	-	-	0
Group II (cataract-untreated)	12	-	-	-	12	all 12 (100%)
Group III (cataract-treated)	12	8	4	-	-	4 of 12 (33.3%)

Group I rat pups received only saline; Group II rat pups received only selenite; Group III rat pups received selenite and rutin hydrate. The degree of opaciﬁcation was graded as follows: 0 = normal transparent lens; + = initial sign of nuclear opacity involving tiny scatters; ++ = partial nuclear opacity; +++ = mature nuclear opacity.

### Results of biochemical analysis

#### Catalase

The mean activity of CAT (expressed as mmol H_2_O_2_ consumed/min/mg protein) in lenses of Group II (3.77±0.36) was significantly (p<0.05) lower than that in lenses of Group I (7.37±0.87) and that in lenses of Group III (6.36±0.52). The mean catalase activity in the lenses of Group III was significantly (p<0.05) lower than that in Group I rat lenses (Table 2).

**Table 2:** Activities of antioxidant enzymes in lenticular samples from rat pups.

**Enzymes (unit of activity)**	**Group I (Normal)**	**Group II (cataract-untreated)**	**Group III (cataract-treated)**
Catalase (mmol H_2_O_2_ consumed/min/mg protein)	7.37±0.87	3.77±0.36^a,b^	6.36±0.52^a^
Superoxide dismutase (unit/min/mg protein)	2.99±0.64	1.95±0.62^a,b^	2.11±0.79^a^
Glutathione peroxidase (µmol glutathione oxidized/min/mg protein)	37.72±7.03	27.55±8.79^a,b^	28.82±7.79^a^
Glutathione-S-transferase (µmol of CDNB conjugate with GSH/min/mg protein)	5.86±0.75	3.60±0.42^a,b^	4.73±0.72^a^
Glutathione reductase (nmoles of NADPH oxidized/min/mg protein)	0.29±0.03	0.23±0.02^a.b^	0.27±0.01^NS^

All values are expressed as mean ± SD of six determinations. Group I: Rat pups received only saline. Group II: Rat pups received only selenite. Group III: Rat pups received selenite and rutin hydrate. ^a^Value significantly different (p<0.05) from Group I value. ^b^Value significantly different (p<0.05) from Group III value.

#### Superoxide dismutase

The mean activity of SOD (expressed as units/min/mg protein) in Group II rat lenses (1.95±0.62) was significantly (p<0.05) lower than that in lenses of Group I (2.99±0.64) and that in lenses of Group III (2.11±0.79). The mean activity of SOD in lenses of Group III was significantly (p<0.05) lower than that in Group I rat lenses (Table 2).

#### Glutathione peroxidase

The mean activity of Gpx enzyme (expressed as µmol glutathione oxidized/min/mg protein) was significantly (p<0.05) lower in Group II (27.55±8.79) lenses than that in lenses of Group I (37.72±7.03) and Group III (28.82±7.79). The mean activity of Gpx in Group III rat lenses was significantly (p<0.05) lower than that in lenses of Group I (Table 2).

#### Glutathione S-Transferase

The mean activity of GST (expressed as µmol of CDNB conjugate with GSH/min) was significantly (p<0.05) lower in Group II (3.60±0.42) rat lenses than that in lenses of Group I (5.86±0.75) and Group III (4.73±0.72). The mean activity of GST in Group III rat lenses was significantly (p<0.05) lower than that in Group I rat lenses (Table 2).

#### Glutathione reductase

The mean activity of GR (expressed as nmol of NADPH oxidized/min/mg protein) was significantly lower (p<0.05) in Group II rat lenses (0.23±0.02) than that in Group I rat lenses (0.29±0.03) and Group III rat lenses (0.27±0.01). However, no significant differences were observed between the mean activity of GR in lenses of Group I and Group III (Table 2).

### Effect on reduced glutathione and malondialdehyde levels in hemolysate and lenses

The mean GSH levels in hemolysate (446.98±48) and lenses (5.22±0.29) of Group II were significantly (p<0.05) lower than that in Group I(hemolysate [782.01±89] and lens [8.39±0.56]) and Group III (hemolysate [678.84±85] and lens [7.33±0.87]). The mean GSH levels in hemolysate and lenses of Group III were significantly (p<0.05) lower than the corresponding values in Group I (Table 3).

**Table 3:** Mean level of reduced glutathione in samples of hemolysate and lenses from rat pups.

**Groups**	**GSH in hemolysate (nmoles/g Hb)**	**GSH in lenses (µmoles/g wet weight)**
Group I	782.01±79	8.39±0.56
Group II	446.98±48^a,b^	5.22±0.29^a,b^
GroupIII	678.84±67^a^	7.33±0.45^a^

All values are expressed as mean ±SD of six determinations. Group I: Rat pups received only saline. Group II: Rat pups received only selenite. Group III: Rat pups received selenite and rutin hydrate. In the table, GSH represents reduced glutathione and Hb represents hemoglobin. ^a^Value significantly different (p<0.05) from Group I value. ^b^Value significantly different (p<0.05) from Group III value.

The mean concentrations of MDA in hemolysate (384.2±70) and lenses (90.29±5.08) of Group II were significantly (p<0.05) greater than the mean concentrations in Group I (hemolysate [286.88±84] and lens [60.13±7.94]) and Group III (hemolysate [307.09±57] and lens [74.23±12.52]). The mean concentrations of MDA in hemolysate and lenses of Group III were significantly (p<0.05) greater than that in Group I (Table 4).

**Table 4:** Mean concentration of malondialdehyde in samples of hemolysate and lenses from rat pups.

**Groups**	**MDA in hemolysate (nmoles/g Hb)**	**MDA in lenses (nmoles/g wet weight)**
Group I	286.88±84	61.23±2.94
Group II	382.79±50^a,b^	90.29±3.08^a,b^
Group III	337.09±72^a^	74.23±1.92^b^

All values are expressed as mean ±SD of six determinations. Group I: Rat pups received only saline. Group II: Rat pups received only selenite. Group III: Rat pups received selenite and rutin hydrate. In the table, MDA represents malondialdehyde and Hb represents hemoglobin. ^a^Value significantly different (p<0.05) from Group I value. ^b^Value significantly different (p<0.05) from Group III value.

## Discussion

Currently, surgical removal of the opaque lens is the standard treatment for human cataracts; however, because the number of individuals who require surgery far exceeds the number of operations being performed, attempts have been made to develop molecules that prevent or delay formation of senile cataracts. Human cataracts are the result of a multifactorial disease associated with several risk factors. Oxidative stress is believed to play a key role in cataractogenesis, and augmentation of the antioxidant defenses of the lens has been shown to prevent or delay experimental cataracts [3].

Protection of the lens from oxidative damage, and hence maintenance of transparency, is afforded by antioxidant systems that consist of non-enzymatic and enzymatic components. Human senile or experimental cataract formation is associated with a progressive decrease in lenticular GSH [35,36]. Therefore, it has been suggested that GSH plays an important role in maintaining lenticular function and transparency by protecting sulfhydryl groups from oxidation [37,38]. The putative importance of oxidative stress in the pathogenesis of cataracts, and in aging in general, has focused attention on antioxidant nutrients.

We have previously reported that an ethanolic extract of the oyster mushroom *P. ostreatus,* could prevent cataractogenesis in an animal model of selenite-induced cataracts. A comparatively high content of chrysin (40 mg/100 g) and rutin (31.2 mg/100 g) was recently observed in an ethanolic extract of *P. ostreatus,* and the authors suggested that these flavonoids possibly contributed to the antioxidant activity of the extract [20]. Rutin modulates a number of biological functions, and exhibits anti-inflammatory and anti-microbial activities [39]. The ability of rutin to scavenge free radicals and to inhibit lipid peroxidation has been reported in conditions of streptozotocin-induced oxidative stress [40]. In addition, rutin has also been reported to play a positive role in carbohydrate metabolism [41], and to elevate the antioxidant status in diabetic rats [42].

In the present study, the observations made by gross morphological examination of the lenses in 30-day-old Wistar rat pups appear to suggest that rutin is able to significantly retard selenite-induced cataractogenesis in these animals. At the end of the study period, all the rat pups that had received only selenite (Group II) were found to have developed a dense nuclear opacity in the lens of each eye, whereas only 33.3% of pups that had received selenite and been treated with rutin (Group III) were found to have mild lenticular opacification in each eye (Table 1; Figure 1A-C). The other 66.7% of pups in that group had clear lenses in both eyes, as in normal pups (Group I).

Antioxidant enzymes are able to catalytically remove free radicals and other reactive species. A wide array of enzymatic antioxidant defenses exists, including SOD, CAT, and Gpx. Superoxide dismutases are present in all eye tissues [43] and are specific for catalytic removal of superoxide. SOD converts superoxide to H_2_O_2_. CAT catalyzes the two-electron dismutation of hydrogen peroxide into ground-state oxygen and water. Gpx and CAT are present in all parts of the eye. Gpx is the predominant GSH-consuming enzyme and the Gpx family uses GSH as a cofactor to destroy hydrogen peroxide and lipoperoxides. Glutathione reductase is the rate-controlling enzyme of the glutathione redox cycle [44], and the intracellular level of GSH is maintained by GR by preserving the integrity of cell membranes and by stabilizing the sulfhydryl groups of proteins [45]. Glutathione S-transferases are a typical multifunctional enzyme, which plays a role in the hydrophobic compounds as a thioltransferase-like redox regulator [46].

In the present study, the mean activities of CAT, SOD, Gpx, GST, and GR were found to be significantly lower in the lenses of cataract-untreated (Group II) rat pups than that in normal control (Group I) rat lenses (Table 2). Lowered activities of these enzymes in selenite-induced cataractogenesis have been well-documented in in vivo experimental animal models [9-11,47]. However, in lenses treated with rutin hydrate in the present study, the mean activities of antioxidant enzymes were significantly higher than the values in rat pups with untreated cataracts (Table 2).

GSH, a ubiquitous essential tripeptide thiol (*L-γ-*glutamyl*-L-*cysteinyl*-*glycine), is a vital intra- and extra-cellular protective antioxidant against oxidative stress. It contains a side chain sulfhydryl (*-*SH) residue that enables it to protect cells against oxidants [48]. Therefore, maintenance of GSH is known to be vital for lenticular transparency. In the present study, the mean level of GSH was found to be significantly lower in the lenses of cataract-untreated (Group II) rat pups than that in normal (Group I) rat lenses (Table 3). A progressive decrease of GSH in the lens has been found to be associated with experimental cataract formation or human senile cataract formation [35,36]. A depletion in GSH content in post-mitotic tissues has also been found to occur during aging, possibly due to enhanced oxidative damage due to free radicals [49]. In the lenses of cataract-treated rat pups (Group III) in the present study, the mean level of GSH was found to be increased (relative to Group II rat lenses; Table 3), with an accompanying increase in the mean activities of GR and GST.

Lipid peroxidation is a free-radical mediated propagation of oxidative insult to polyunsaturated fatty acids, involving several types of free radicals; termination occurs through enzymatic means or by free radical scavenging by antioxidants [50]. Increased lenticular MDA may be the result of increased lipid peroxidation of the lenticular cell membranes, or may represent the consequence of migration of MDA from the readily peroxidizable retina or from the central body compartment [51-55]. In the present investigation, such a disruption of membrane lipids possibly accounted for the observed increase in MDA levels in the lenses of cataract-untreated (Group II) rat pups when compared to normal (Group I) rat pups (Table 4). The observed lower mean MDA level in the cataract-treated (Group III) group (relative to Group II values) suggests that rutin hydrate possibly preserved the structural integrity of the lens, thereby preventing lenticular opacification. These data corroborate the findings of earlier investigators [9,10,14].

From the results obtained, it appears that rutin prevents selenite-induced cataractogenesis in Wistar rat pups. Morphological assessment showed that the damage caused by selenite-induced cataractogenesis was attenuated by the administration of rutin. The results of biochemical evaluation suggest that rutin prevents cataractogenesis by enhancing the activities of antioxidant enzymes and functions of the redox system, and by reducing the extent of lipid peroxidation.

A question may arise regarding the relevance of the results of the present study, done in Wistar rats, to the situation in humans. In this context, the following points should be considered. In a study on the intake of flavonoids and isoflavonoids in volunteer Japanese women, the average daily intake of rutin was found to be 1.5 mg and that of quercetin (closely related to of rutin) was 8.3 mg [56]; vegetables in the diet were the major source. While this level of intake may seem low, it is important to note that dietary supplements of rutin are commercially available as 500 mg tablets and capsules [57]; the recommended dosage of these supplements is 500 mg taken once or twice daily. Interestingly Boyle et al. [58] reported that when 500 mg rutin supplementation was given daily for 6 weeks to human volunteers, it did not induce any adverse changes in blood chemistry or indices of liver function. Thus, oral intake of 500 mg of rutin (as a supplementary tablet or capsule) per day possibly approximates the intraperitoneal dosage of rutin used in the present study in Wistar rat pups (175 mg/kg body weight per day), although pharmacokinetic studies would be needed to definitively confirm this.
